# Supplementary material for: Ion dynamics at the energy-deprived tripartite synapse
Source: PLoS Comput Biol. 2021 Jun 18;17(6):e1009019. doi: 10.1371/journal.pcbi.1009019 (PMC8244923; doi:10.1371/journal.pcbi.1009019)
Supplement: Attachment — Submitted filename: Kalia.PCOMPBIOL-D-20-01345-0.pdf [file pcbi.1009019.s005.pdf]

PCOMPBIOL-D-20-01345

Title: Ion dynamics of the energy-deprived tripartite synapse

Authors: Kalia M, Meijer HGE, van Gils SA, van Putten MJAM, Rose CA

Overview

Kalia and co-workers present a detailed biophysical model of ion homeostasis at cortical synapses, including astrocytic signaling. Astrocyte-regulated synapses, aka tripartite synapses, represent 50-80% of cortical synapses, but their physiology remains an open matter of active investigation. This study fills in this line, focusing on neuron-glial-based ion homeostasis in setting ischemic conditions, both in the healthy and the aged brain. This is undoubtedly an exciting study, which aims at tracking down biophysical pathways for the complex topic of ion (dys)homeostasis in the brain, and the fact that it takes into account glial signaling makes it one of the few studies in this sense currently available in the literature – and arguably one of the most detailed ones. Accordingly, I deem the manuscript of interest for PLoS Computational Biology. However, I am afraid I cannot recommend it for publication in its current version due to some severe pitfalls. The writing style is generally poor, and manuscript and figures' organization can be vastly improved. Accordingly, I am suggesting significant revisions before re-considering the manuscript for publication.

Suggestions for major revisions

Title:

1. Ion dynamics *at* the energy-deprived tripartite synapse.

Abstract:

I would avoid to keep on referring to your previous model. This is detrimental to the promotion of the novelty of your work. In the abstract accordingly:

1. Drop: "Significantly extending earlier simulations..." start directly from "Our model is based on fundamental biophysical principles...".
2. drop: "As opposed to earlier simulations..." → Our model predicts that...
3. The last sentence is cumbersome; I would rephrase it along the lines of: "Our work provides clinically relevant insights into the neuropil's ability to recover from energy deprivation, with translational relevance for diagnosis and treatment of ischemic strokes."

Introduction:

There are several issues with your current introduction. It would help if you emphasize what is not known and your work's relative contribution, especially putting in relief the link with glia. Generally speaking, your introduction revolves around four concepts: ischemia, volume regulation, energy supply in synaptic transmission, and tripartite synapses. You are failing to put these four elements in a logical rationale.

1. L2-8: It is better to refer to "information transfer at synapses" rather than at tripartite synapses: in fact, you are introducing the concept of tripartite synapses, later on, starting from L30.
2. L44-64: It is not clear the link between ischemia/spreading depression/pathology in general with volume regulation. L56-L64, where you introduce volume regulation through aging...

you need to rewrite this part and link pathology with aging if you aim to keep the material you have at the moment.

3. L9-29: This paragraph is way too technical for an introduction. Consider moving it to Model description, as a separate introductory "Biophysical background" complemented accordingly with more details of your model.
4. L73-82 contains material that can be split between Methods and Discussion. It is not relevant to the introduction.
5. L92-95: Strengthen your message, please: "We believe our results aid in..." → you'd rather make predictions than believing.

Model Description

1. Use a more descriptive section subtitle: e.g., "A model for ion homeostasis at the tripartite synapse."
2. Then consider pre-pending a section in which you clearly explain Figure 1.
3. Accordingly, proceed with the tripartite synapse model and calibrations to follow (three subsections with informative section titles, therefore).
4. I am missing out on your current description of the model how it extends the previous one. So it is probably better to start from a concise description of your formal model and emphasize the new components.
5. I am also jeopardized because you separate between neuronal and synaptic compartments, but if I am not mistaken, form the equations, it seems that the synaptic part is within the neuronal compartment. Please clarify this aspect in your model description and corresponding figures.
6. L133-L153: I do not understand your motivations for eqs. 5-9. I think it would make sense to start by introducing data in Ref. 8 and driving the reader accordingly through your modeling arguments. L139: why should we have "chemical ischemia" in italic?
7. Why your model's fit/calibration in Fig. 3, Na+ traces of astrocytes and neurons have such a different shape from experiments? What contributes to it in your model? Please provide a brief account for it in the text and the figure caption.

Results:

There is some mess with your figures, starting from Figure 6. I have not sorted it out frankly, but it seems that you are missing at least one figure (some hypothetical Figure 9. Your current Figure 9 is what you dub as Figure 7 in the manuscript). Moreover, you are discussing results in a confusing way that does not reflect figures' numbers (e.g., Figure 6B after Figure 7; Figure 7(9) after Figure 8, frankly I got lost). I would advise using the abbreviation ED for "energy deprivation" throughout the text.

1. L159,161, 174: "functional", "fully functional" and "non-functional" astrocyte are ambiguous.
2. L170: I do not understand "override."
3. L172: It seems that your result here depends on the transients' analysis, so I do not understand why you are omitting to show it in the figure.

4. L188: it is a bit hard to catch bistability. As I suggest below, probably transposing your figure improves readability, but the primary notion here that you are failing to state is that before and after ED, you get different steady-state solutions.
5. L208: what is "negligible" for you? Why?
6. L213,219,227: Figure number is missing.
7. L249-251: It is a good result. Link with some relevant reference and re-discuss it in the discussion section too.
8. L279: Why "biophysically unrealistic"?
9. Your claims concerning steady-state values are poorly substantiated based on what you show in Figs. 4, S1, and 5. In Figure 5, for example, the Volume does not seem to be at a steady-state. Improve results and substantiate your claims by improved simulations if necessary.
10. L264-311: I lost you. You are jumping back to Figure 6B, then refer to Figure 7B, very confusing. Rewrite, and rearrange figures accordingly (see also my related suggestions). Noteworthy is that In Fig.6, you consider alpha_e=20% vs. 80%. In Fig.7 instead you refer to alpha_e=20% vs 98%. Please be consistent. Moreover, 98% seems a bit extreme as a case study.
11. L334-348: I am missing Figure 9; hence I cannot comment on this section.

Discussion:

1. Overall, you are failing to say what's new by your model. This is arguably in line with the fact that the research question in the introduction is not clearly stated. Some of the results also appear not to be discussed appropriately, like those in the "Pharmacological Blockers" section, which could be linked with insights on useful drug targets.
2. L391-400: Overtly technical. Simplify and emphasize oscillations instead.
3. L439-456: These limitations can only be briefly mentioned as an extra paragraph appended to the "Relation to other computational models."
4. You should instead replace "Limitations" with "Future directions" or "Open questions." In particular, there are three topics that you have not discussed, but you should briefly mention and refer to: the first is the fact that your ED framework is intrinsically linked with the glutamate-glutamine cycle and possibly with lactate shuttling. Additionally, these processes are inherently linked to bicarbonate ions and pH regulation. It is true that currently, you do not consider pH regulation, but the fact that you are including sodium and chloride homeostasis brings in bicarbonate trafficking too. There are a couple of recent references you may look at De Pitta in Encyclopedia of computational Neuroscience 2020; and Theparambil et al. (Rusakov and Gourine, as senior authors), Nature Communications, 2020.

Equations and notation:

1. E1: the variable q refers to multiple kinetic variables: you should subscript it accordingly and then refer to the supplementary material. Personally, there is no point in introducing ODE systems like 1 if you do not put in relief at least the functional coupling between the relevant variables.

2. You seem to interchangeably use P_{min} and P_min (e.g. eq. 5 and L255 vs. Table 2 or L246). Please be consistent in your notation.

Figures:

Your figures are part of the manuscript that requires the most work. They need substantial improvement, as most of them are lousy in their organization and lack clarity.

1. Figure 1. Improve readability, adding what ions are transported/pass through the different elements. Why is water flux by a different arrow? It is mediated by AQPs in general, which are themselves proteins like the other elements. The shading of pre/peri-synaptic compartments is barely distinguishable. Use "Extracellular Space" instead of ECS.
2. Figure 2. Merge with Figure 1, and also specify ions for all arrows. Use another box to zoom into the synapse model. It is better to redraw this model in a circular fashion (curvilinear arrows) to convey the notion of vesicular recycling better. "F" state variable then is at the bottom center, and from there, you work around the vertices of a pentagon for F, R, N, D, and I, yet using curvilinear connections. Be consistent in your notation: It is either Ca2+ or [Ca2+]n.
3. Figure 3. Use full axes, not mini-axes: this will improve readability and comparison. Use the same scales for both experimental and model data. In this figure, and all the following: there are more letters than A and B in the Latin alphabet. Do not hesitate to use them to label panels in your figures properly. In this figure: rearrange the material vertically: panel A on top center. Then, panel B is in two columns (explain to me what the gray curves are). And then C for Na+ and K+ in the extracellular space. Use the same figure size for these data as in B, arrange Na+ and K+ in separate rows, and ease comparison.
4. Figure 4. Transpose. Rather than two rows, make two columns, and stretch the width of individual plots if possible. Use colored labels with subscripts rather than titles, and for colored axis tick labels, also use colored axes and ticks. There is no point in presenting results that refer to transients if you do not show them. Modify figures accordingly. Also, B plots are all in blocked conditions, and the big red box is hideous. Improve aesthetics, please. Also, use proper axes ticks. You have a massive space after 10 mins, for example. Same case for some of the y-axes. Set 3-5 axis ticks in all axes, please. Append legend at an ideal position for different line colors.
5. Figure 5. Same observations as in Figure 4. Again: each row can be conveniently labeled by a letter, and you can refer to them much more quickly in the text: it saves the reader's time to identify what plot you are referring to by the need to read axes labels/titles.
6. Figures 6A and 6B should be presented separately. 6B could maybe be lumped with your current Figure 9 (for OGD) or 7B together with 8? As I said, this part of your results was not readable. 6A use circled numbers as you do in Figure 8 to refer to point to left plots and avoid crowding of unnecessary arrows. Also, I would flip the panels: heatmap on the left and plots on the right. Associate title with color bar. I do not understand arrows in axis labels. I do not understand the "Extreme energy deprivation" label. Figure 6B: the middle table is not explained. If you retain it, put x label on top. Do we need arrows?
7. Figure 7: This figure is entirely unreadable. Again rearrange material vertically. Figure 7B seems instead related to figure 8. Concerning your bifurcation plots: Avoid zooming inboxes,

and instead enlarge main axes to make diagrams larger and more readable. Choosing different colors is a debatable idea, but if you keep them, append legend. Plot trajectories in separate plots and not within the bifurcation diagram. I am also not sure I understand those trajectories. It seems that P_min is changing from your plots, but in the caption, P_min is said to be fixed. Regardless, it is confusing to map a trajectory on a bifurcation diagram, where the x-axis is a parameter. Make two new plots for P_min(t) or whatever you are changing in time to obtain the Volume (t) in the bottom plots.

8. Figure 8: please mark B1 and B2 as boxes accordingly, and append on the bottom of A, as two panels of half-width the x-axes in A. Merge with current Figure 7B (and perhaps 6B). Again, the material as I got it seems messed up.

Figure/Table captions.

1. Figure 1. The largest *ATP consume* in the presynaptic neuron, and the astrocyte is *by NKA*.
2. Figure 2. This is not a caption. Provide essential model description details accordingly in the new version (merged with figure 1).
3. Figure 6. you compare "large" ECS with "realistic" ECS. I do not understand your concept of "realistic." Try to be as much as possible consistent with your text: if you use physiological vs. pathological, for example, or normal vs. swelling/shrunk, then adopt the same terminology throughout the whole manuscript.

References:

L 41: reference 20 should be replaced by De Pitta, Chapter 10 in Computational Glioscience 2019.

Bibliography:

Please review your bibliography entries: there is an eclectic style: some entries are in capitalized for each word vs. others that use only sentence's capitalization. Be consistent in reference/bib style, please.

Spelling and grammar issues:

I would advice to use Grammarly for a thorough spell/grammar check upon re-submission. Please do so. In addition mind the following typos and grammar errors:
L33: inter-relationship → interaction;
L38: elucidated with… calibrated with… → by … by…;
L105-108: channels/cotransporters and currents/fluxes is confusing. Simplify accordingly.
L109: A ~~short~~ summary.
L123: neuronal ~~synaptic~~ compartment.
L133+ (last line): The U-shaped function I_NKA… it Is actually I_block that is U-shaped…
L197: unnecessary italic.
L219,382: Further*more*, …
L220-221: cell swelling is observed (approximately…).
L225: This is in constrast to the scenario discussed in Fig. 5A for… where…
L227: S2 Fig… correct accordingly.
L229: depth → amount
L231: … the pump’s strengh.
L232: ~~Using our model,~~ *W*e explore…

L253: wrong figure reference.

General recommendations:
In the next version, please lump all figure captions and tables to the end, providing the main body of the manuscript without interruptions.
Be careful when you re-submit. The impression I got from your current manuscript is that you submitted it quickly and without too much care, as if you were coming from rejection from another journal, and you promptly rearranged the material for PCB. Whether or not this is the case, it does not play in your favor to submit a manuscript to PCB with messed-up figure's numbers and missing figures, as is the case of the present version.
