# Supplementary material for: Ion dynamics at the energy-deprived tripartite synapse
Source: PLoS Comput Biol. 2021 Jun 18;17(6):e1009019. doi: 10.1371/journal.pcbi.1009019 (PMC8244923; doi:10.1371/journal.pcbi.1009019)
Supplement: Attachment — Submitted filename: response.pdf [file pcbi.1009019.s006.pdf]

To:

The Editorial Board
PLOS Computational Biology

University of Twente
Drienerlolaan 5
7522NB Enschede
The Netherlands

January 12, 2021

In response to decision: PCOMPBIOL-D-20-01345

Dear Editors,

We thank all reviewers for their valuable comments. Please find a detailed point-by-point rebuttal below. The comments made by the referees are within grey blocks, whereas our responses are in normal style. Excerpts from the manuscript are in italic. Kindly do note that line references in our responses are with respect to the new submission.

REFEREE REPORT(S):

Referee 1 (Reproducibility report)

We thank the referee for their efforts in successfully reproducing of the simulations we show in the paper.

Referee 2

The manuscript by Kalia and colleagues titled "Ion dynamics of the energy-deprived tripartite synapse" develops a model of the tripartite synapse in physiology and in periods of energetic stress. The manuscript is extremely well written and very clear. It represents a commendable effort by the authors to develop an almost exhaustive model of ion transport at the tripartite synapse, and is a perfect fit for this journal in this reviewer's opinion. The authors proceed to compare and analyse the dynamics of the system when facing the challenge of energy deprivation of various durations and severity. The work is a very nice computational biology study with broad relevance in neuroscience. This reviewer's enthusiasm is however tempered slightly by some issues that could be important for the validity of the results.

We thank the reviewer for their appreciation of the work and the critical points raised.

Major:

- Both the time and concentration scales in Fig. 3B are different, which makes it difficult to evaluate how well the model reproduces experimental data after calibration. Please adjust these.

  We agree that the time and concentration scales are different, and we have adjusted the concentration scale of the astrocyte Na^+ trace to match the experimental data. However, for the rest of the traces, we prefer to keep the scales as they are for illustration purposes. Note that the traces qualitatively match the experimental data, which is essential to the main results of the manuscript. We have added statements, describing the qualitative difference between astrocytic and neuronal Na^+ transients in the simulation, to the manuscript (see below).
  ' *As details about the change of NKA activity resulting from chemical ischemia used in the experiments are not completely known, we observe some...*'

- There seems to be an issue with the potassium and sodium dynamics in the model, which does not reproduce or capture experimental results accurately. This is apparent in Fig. 3B again, where the undershoot after a period of energy deprivation present in experimental data is not captured by the model. Similarly, we would expect sodium in astrocytes to go up when the synapse is activated. The model predicts it goes down instead. The authors cite their own work but that paper actually says: "Astrocyte sodium concentration increased by up to 8.5 mM, which could be followed by an undershoot below baseline." and the text of the present manuscript says: "In our simulation, astrocyte Na+ decreases in response to neuronal burst firing, which is also observed experimentally under conditions of recurrent network activity and activation of astrocyte NKA by increases in extracellular K+ [41]. Notably, when Na+-dependent glutamate uptake or influx through NMDA receptors override the NKA-induced export of Na+, a net increase in astrocyte Na+ is induced [41]." There seems to be an important discrepancy here between what the model does when the synapses is activated and what experiments observe. This reviewer thinks that this is due to how glutamate recapture is implemented in the model. Figure 2 suggests for instance that this only happens in neurons. The authors do say in line 540 that 90% of the reuptake happens in astrocytes however, which is probably correct experimentally and roughly matches the distribution of these transporters as observed in the Barres transcriptome. I believe the authors should make sure that the 90% glutamate uptake is true overall and not only at baseline (see Table 5). That might solve this issue.

We thank the referee for this critical appraisal and the suggestions. The major sodium and potassium traces from the model in response to energy deprivation as shown in Figure 3 reproduce experimental data fairly well. We agree that the scale is different, but the responses are of similar magnitude.

The 9:1 ratio of astrocyte vs neuronal glutamate uptake via EAATs are maintained throughout the simulation. Regarding the astrocytic sodium dynamics in response to synaptic activation, we agree that this is a limitation of the current model, but at the same time would like to point out that the paper cited explicitly also describes and illustrates astrocytes which undergo a brief increase in Na^+ only, followed by a longer undershoot during burst activity of neurons (see also below). We have tried a variety of modifications, both model parameters and adding currents such as NBC and NMDA to the astrocyte. None of these yielded a plausible response as they typically violated baseline conditions for the leak currents. All in all, as the reviewer points out, this discrepancy might well be caused by the uptake mechanism for glutamate. For the glutamate recycling, however, we are following current literature. Adding the glutamate-glutamine cycle to the model requires many more experimental results, and will result in a paper of its own.

Given the good qualitative match to experimental data for the bulk ions during energy deprivation, and that the glutamate response is not the major focus, we now discuss these limitations explicitly.

Specifically, we now add the following lines to the Discussion, **lines 386-395**:
"*However, upon neuronal stimulation, astrocyte* Na^+ *decreases. Earlier experimental work performed in brain tissue slices [43] has shown that most of astrocytes undergo an increase in intracellular* Na^+ *during neuronal bursting activity. In about a third of cells, however, the increase was rather brief and was followed by an undershoot in* $[Na^+]_i$. *This undershoot could be mimicked by increasing extracellular* K^+, *indicating that it was due to activation of astrocyte NKA. Moreover, combined addition of glutamate and high* K^+ *caused a biphasic increase-decrease in some cells, indicating that both processes - activation of NKA resulting in an export of* Na^+ *and activation of glutamate uptake resulting in an import of* Na^+ *- counteract each other. Notably in our model, NKA-induced export of* Na^+ *is higher than EAAT-mediated* Na^+ *uptake in astrocytes.*".

We also mention in the Discussion, in the new section 'Future directions' **line 501-505**:
'*Moreover, by introducing pH regulation, also the sodium-bicarbonate cotransporter (NBCe1) [58] and/or the sodium-proton exchanger (NHE) could be incorporated. These mechanisms will increase* Na^+ *transport into astrocytes, and might result in a net increase in astrocytic* Na^+ *with physiological stimulation, improving the current model.*'

To conclude, we appreciate the validity of this comment, but believe it requires substantially more work to solve resulting in a significantly extended model incorporating more Na^+ transport processes across the membranes. Importantly, it does not affect the main message of the work regarding recovery, vulnerability and extracellular spaces, and thus remains as a limitation of the current modeling approach.

- It is unclear to this reviewer how the volumes of the cells are modelled. Eq 1 in page 4 does not seem compatible with Eq. Mod. 2 in page 23. Please clarify.

The model equations describe evolution of ions N_X^i and volumes W_i for the various compartments i. The various volumes are modeled in accordance with Eq.1 (after **line 119**) where λ_i is given by Eq. 44 . Let us stress that the volumes of the 'synaptic compartments' W_c, W_{ps} and W_{pap} are kept constant. We agree that the presentation could be more transparent. Therefore, we added Eq. 44 to better explain the relation between Eq. 1 and Eq. Mod. 2. Also, we changed Table 4 and 5 to show values of $L^i_{H_2O}$ instead of λ_a. We hope this clarifies this concern.

Minor:

- Some figure references are missing around line 213.

  Thank you for pointing this out. We have changed the figure references.

- Figures 7 and 8 appear to be swapped, or their legends are.

  We apologize, this was an unfortunate mistake from our side. This has been corrected.

- The authors might want to check the model of brain energy metabolism published by the Magistretti group in 2015 (PLOS Comput Biol) for the sake of completeness.

  Thank you for this suggestion. We now discuss this work in our new Discussion section 'Future directions' **lines 498-501**, with the following lines:

  '*The first predictions from our model about glutamate transients can benefit from models such as [55] to include the glutamate-glutamine cycle, which is critical to dynamics of neurotransmitter replenishment.*'

Referee 3

Dear Authors,

It was very interesting to read your manuscript "Ion dynamics of the energy-deprived tripartite synapse". I have some comments which should be addressed.

We thank the reviewer for their appreciation of the work and the critical points raised.

Introduction:

- Page 2, line 21: Since you model the volume changes, it would be helpful to explain the mechanism of cell swelling in 1 or 2 sentences.

  We agree that this explanation is important, and actually provided it in the original submission at **line 211-213**, where we wrote *Thus, intracellular Cl^- increases to preserve electroneutrality [46]. The resulting osmotic imbalance leads to water influx, i.e., cell swelling [12].*. We prefer to keep it at that point in the text as there we describe the dynamics of swelling in detail.

- P2, l 40: Please include some review articles here: e.g., doi.org/10.3389/fncom.2018.00014, doi.org/10.1016/j.brainresbull.2017.01.027, book doi.org/10.1007/978-3-030-00817-8

  We thank you for this valuable suggestion, and we have added these review articles accordingly.

Model Description:

- P 5, equation 3: Describe C_W and C_X in the text.

  We describe these constants by adding the following line after Eq. 3, (**line 134-135**):

  '*Here, the constants C_W and C_X are the total volume and total molar amount of ions present in the system respectively.*'

- P 5, Figure 2: Please extend the figure caption. In the figure itself, what does the bracket mean in "R1: Vesicle bound -1 Ca2+)" (same for R_2)?

The caption for Figure 2 was extended to describe the glutamate recycling scheme. Also, the caption explains the meaning of $R_{1,2,3}$ as the number of calcium ions bound to a vesicle. The new caption now reads:

'*Glutamate recycling scheme, inspired by combining vesicle-based models from [41] and [42]. (Left) Closer view of the model scheme at the synapse and (Right) the glutamate recycling scheme. Inactive neuronal intracellular glutamate (I) moves to the depot (D) from where it is packed into vesicles which pass through five stages (N,R,R1,2,3) before they are released into the synaptic cleft (F). These stages have fast time-constants that depend on intracellular* Ca^{2+} *concentration. The stages* R_i *correspond to vesicles that are bound by i* Ca^{2+} *ions. The time-constants change when there is influx of* Ca^{2+} *in the presynaptic terminal in response to membrane depolarization. Released glutamate in the cleft can be taken up by astrocytes or back to neurons using excitatory amino acid transporters (EAATs) or leak channels, thereby recycling the released neurotransmitter.*'

- P 5, l 134: For the reading flow, it is helpful if you shortly describe f^i_{NKA} at this point and then refer to Materials and Methods (as done).

  A description of the function f^i_{NKA} was added, just after equation 4, below figure 2 on page 6 (line numbers do not appear), which reads:

  '*The expression* f^i_{NKA} *describes the inward NKA current. It depends on neuronal* Na^+ *concentration and extracellular* K^+ *concentration. Details can be found in Materials and Methods.* '

- P 6, l 139: In my opinion, the sentence is ambiguous. It is not clear if the whole recording is 2 minutes long or the ischemic part.

  The whole experiment lasts longer, only the perfusion with metabolic inhibitors is 2 minutes long. This 'chemical ischemia' induces a transient drop in intracellular ATP that lasts longer than 2 minutes. Thus we changed the summary of these experiments to (**line 147-148**):

  "*In these experiments, metabolism is inhibited by exposing cells to 'chemical ischemia' for 2 minutes.*"

- P 6, Figure 3: Do you have an explanation for why you have a sharper peak in the neuronal Na+ and why your modeled peaks are at the end of the ischemia period while they are earlier in the experiments?

  Thank you for your question. We indeed have an explanation for this qualitative difference in Na^+ calibration traces. In order to explain it, we added the following line after the end of sentence in **lines 152-160**:

  '*As details about the change of NKA activity resulting from chemical ischemia used in the experiments are not completely known, we observe some differences in neuronal and astrocyte* Na^+ *gradients during the onset of ED. In neurons, the presence of voltage-gated* Na^+ *channels that are activated upon membrane depolarisation causes a fast and strong* Na^+ *influx. In contrast, the model for astrocytes does not have any fast, channel-mediated* Na^+ *influx pathway, resulting in lower* Na^+ *rise. Thus, we characterize the chemical ischemia experiments from [8] with the parameter values* $\alpha_e = 80\%$, $P_{\min} = 50\%$ *and* $P^n_{NKA} = 86.4$ *pA, and use them for further simulations presented in this work.*'

- For a modeler, it would be interesting to know how you have done the fitting process.

  We now specify that most of the parameters are taken from previous work, while only those parameters that we introduce here (related to ECS and pump strength) were manually tuned. Thus, the following lines were added at **lines 145-147**:

  '*All the parameters in this model have been sourced from previously published work. However, we set the parameters* α_e, $P_{\min}$, $P^n_{NKA}(= P^a_{NKA})$ *to fit the experimental traces in [8] using an empirical parameter search.*'

Results

- P 7, l 159: I suggest to exchange" Experiments" by" Simulations" to avoid confusion.

  Line 159 (now line 161) was changed accordingly.

- P 8, l 188-191: Please refer to the corresponding figure again .

  Figure reference was added.

- P 8, Figure 5 caption: What means “gradually reduced” and “restored back”? Please specify if this was done over a time period or instantaneously.

  The gradual reduction and restoration is identical to Fig.3-A. We have added this to the caption now, to make it clear. Thus the last line of the caption reads:

  ‘*Shaded grey areas correspond to the period where* Na^+/K^+*-ATPase (NKA) activity is gradually reduced to* P_{min} *and restored to baseline, identical to Fig. 3-A.*’

- P 9, l 219: The figure number is confusing.

  The reference was to the supplementary Figure S2, which is now resolved.

- P 9, l 242: I suggest to change the order of $\alpha_e = 20\%$ and $\alpha_e = 80\%$ in this line since the text afterward and the figure start with $\alpha_e = 80\%$.

  This was changed accordingly.

- Figure 7: Please be aware that you had swapped Figures 7 and 9 when you uploaded them.

  We thank the reviewer for pointing this out, the figures and the captions are now updated in the correct order.

- Figures 6 and 8: I suggest to put Figure 6-A and 8-A together in one. It seems more logical to me.

  The reviewer is correct that Fig. 8 expands upon Fig. 6-B1. However, in Fig. 8 we consider events and responses post energy deprivation, for which this little part of the simulation has to be repeated for clarity. Therefore, we prefer to keep it as it is.

- Figure 6-B: Please use the same x- and y-axes for the subfigures. It would be easier to compare them.

  Thanks for pointing this out, we have updated these subfigures.

- P 11, l 305-306: Please remove the sentence ”In Fig. 6-B.3,4, P^{scale}_{NKA} is set to 2.” You have mentioned it already in line 297.

  The sentence was removed.

- P 12, l 348: I suggest to add the not shown data to the supplement.

  We have added this to the supplementary material (S3 Fig.), and refer to it, see **lines 362-364**.

Discussion:

I found the following interesting review article: ”Molecular mechanisms of K+ clearance and extracellular space shrinkage—Glia cells as the stars ” (https://doi.org/10.1002/glia.23824). Maybe you find more evidence in the article that supports your findings.

Thank you for pointing this out. The article raises several interesting points and we refer to this elegant review in our paper in **lines 383-385**. The line now reads:

‘*As expected [47], astrocytic dysfunction in our simulations also results in disruption of* K^+ *homeostasis and cessation of synaptic transmission, even in conditions with initially preserved neuronal function.*’

Conclusions:

P 14, l 459ff.: Please be more specific on your findings.

We added the following lines to the end of the Conclusion (**lines 507-518**):

‘*We introduced a model of the tripartite synapse that describes ion and volume dynamics at synaptic and somatic levels. The model faithfully reproduces biological observations and identifies potential treatment targets to limit permanent synaptic failure in clinical conditions characterized by temporary energy failure. The model predicts that synapses surrounded by smaller extracellular spaces are more vulnerable to ischemia and that differential expression of baseline NKA may explain regional differences in ischemic vulnerability. Further, it predicts that blocking neuronal voltage-gated* Na^+ *and* K^+ *channels rescues the synapse from the pathological state post transient ED. Moreover, the model allows us to include additional processes and combine with more models from current literature to create a mathematical description of critical events concerning synaptic regulation in physiological and pathological conditions at the tripartite synapse.*’

Material and methods: P 15, equation 10: I could not find the description of z_X in this section.

The description of z_X has been added immediately after its introduction in **Eq. 10**. The phrase reads (**page 21, after Eq. 10**):
'*where* $i = \{n, a\}$ *and* z_X *is the valence of the ion/species* X.'

Supplementary Figure S1: What does the "+1.8" mean in the right y-axis label of the Ca2+ concentration? Please specify in the caption.

The '+1.8' corresponds to the factor on the y-axis, which is to be added to the scale shown after multiplying with 10^4. As suggested, we have added this to the caption as follows:

'*The green extracellular calcium trace has an offset of 1.8 mM.*'

Referee 4

Kalia and co-workers present a detailed biophysical model of ion homeostasis at cortical synapses, including astrocytic signaling. Astrocyte-regulated synapses, aka tripartite synapses, represent 50-80% of cortical synapses, but their physiology remains an open matter of active investigation. This study fills in this line, focusing on neuron-glial-based ion homeostasis in setting ischemic conditions, both in the healthy and the aged brain. This is undoubtedly an exciting study, which aims at tracking down biophysical pathways for the complex topic of ion (dys)homeostasis in the brain, and the fact that it takes into account glial signaling makes it one of the few studies in this sense currently available in the literature – and arguably one of the most detailed ones. Accordingly, I deem the manuscript of interest for PLoS Computational Biology. However, I am afraid I cannot recommend it for publication in its current version due to some severe pitfalls. The writing style is generally poor, and manuscript and figures' organization can be vastly improved. Accordingly, I am suggesting significant revisions before re-considering the manuscript for publication. Please refer to the attached PDF for suggestions for editing. Thank you for your consideration.

We thank the reviewer for their appreciation of the work and the critical points raised.

Suggestions for major revisions
Title:

1. Ion dynamics at the energy-deprived tripartite synapse.

Thank you for this suggestion. We agree with the recommendation, and have decided to change the title accordingly.

Abstract

I would avoid to keep on referring to your previous model. This is detrimental to the promotion of the novelty of your work. In the abstract accordingly:

1. Drop: 'Significantly extending earlier simulations. . . ' start directly from 'Our model is based on fundamental biophysical principles...'
2. drop: 'As opposed to earlier simulations. . . ' → Our model predicts that...
3. The last sentence is cumbersome; I would rephrase it along the lines of: 'Our work provides clinically relevant insights into the neuropil's ability to recover from energy deprivation, with translational relevance for diagnosis and treatment of ischemic strokes.'

We agree with the reviewer's recommendation to focus more on the novelty and not referring to previous work. The above three changes were made to that regard.

Introduction:

There are several issues with your current introduction. It would help if you emphasize what is not known and your work's relative contribution, especially putting in relief the link with glia. Generally speaking, your introduction revolves around four concepts: ischemia, volume regulation, energy supply in synaptic transmission, and tripartite synapses. You are failing to put these four elements in a logical rationale.

We thank the reviewer for these valuable suggestions. We have modified several parts of the introduction, and we hope the flow is clearer now.

1. L2-8: It is better to refer to 'information transfer at synapses' rather than at tripartite synapses: in fact, you are introducing the concept of tripartite synapses, later on, starting from L30.

   The phrase has been appropriately changed.

2. L44-64: It is not clear the link between ischemia/spreading depression/pathology in general with volume regulation. L56-L64, where you introduce volume regulation through aging... you need to rewrite this part and link pathology with aging if you aim to keep the material you have at the moment.

   Thank you for this suggestion. We, however, disagree with this comment. There are two paragraphs between lines 44 and 64. The first argues that the pathophysiology of ischemic stroke in the context of ion/volume regulation and synaptic transmission is not holistically explored in models present in current literature. Volume dysregulation is already introduced in line 21 as an outcome of ion accumulation (Na^+ and Cl^-). The second paragraph describes volume changes with respect to aging. These two paragraphs are thus independent. Therefore, we prefer to stick with the current structure of the text.

3. L9-29: This paragraph is way too technical for an introduction. Consider moving it to Model description, as a separate introductory 'Biophysical background' complemented accordingly with more details of your model.

   We appreciate the suggestion and we have considered this option. Eventually, however, we decided to keep it as a part of the introduction. While indeed somewhat technical, we think it is digestible for the intended audience of Plos Comp Biology; we did modify some parts to improve legibility.

4. L73-82 contains material that can be split between Methods and Discussion. It is not relevant to the introduction.

   Thank you for the suggestion. We agree that this was phrased as a discussion point, but we prefer to keep this paragraph as it gives a brief outlook of the modeling novelty vis-a-vis previously published work, and is thus essential to the Introduction. To present this material properly, we adapted the first line **line 73-74** to:

   '*We propose a novel model combining elements from existing models into a comprehensive, biologically realistic description.*'

5. L92-95: Strengthen your message, please: 'We believe our results aid in...' → you'd rather make predictions than believing.

   The line 'We believe our results...' has been changed to **line 92-93**:

   '*Our results aid in our understanding of the early events following energy depletion.*'

Model Description

1. Use a more descriptive section subtitle: e.g., 'A model for ion homeostasis at the tripartite synapse.'

   The section title was changed according to the suggestion made.

2. Then consider pre-pending a section in which you clearly explain Figure 1.

   We appreciate the suggestion. We, however, think that at this stage of the text the main elements of the figure have been introduced. Hence, it serves as an illustrative reference of what the model contains regarding geometry and channels. The detailed explanation of each model component is given in the supplementary text.

3. Accordingly, proceed with the tripartite synapse model and calibrations to follow (three subsections with informative section titles, therefore).

   We have introduced a new subsection 'Model equations'. The section 'Simulations and model calibration' was converted to a subsection.

4. I am missing out on your current description of the model how it extends the previous one. So it is probably better to start from a concise description of your formal model and emphasize the new components.

   After the sentence in line **98**, we added:

   '*The model in [11] contained a single neuronal compartment in an infinite extracellular space, whose volume is regulated by changes in osmolarity.*'

5. I am also jeopardized because you separate between neuronal and synaptic compartments, but if I am not mistaken, form the equations, it seems that the synaptic part is within the neuronal compartment. Please clarify this aspect in your model description and corresponding figures.

Thank you for indicating this. To clarify, we added the following text (**lines 106-110**):

'*We further assume that* Ca^{2+} *and glutamate are confined to the presynaptic terminal, the perisynaptic astrocyte process and cleft. The concentrations of ions* Na^+, K^+*and* Cl^- *are assumed to be the same in the synaptic compartments as in the somatic ones. This allows us to work with both, small molar changes of* Ca^{2+} *and glutamate and large* Na^+, K^+*and* Cl^- *gradients across the somatic membranes, within the same compartmental framework.*'

6. L133-L153: I do not understand your motivations for eqs. 5-9. I think it would make sense to start by introducing data in Ref. 8 and driving the reader accordingly through your modeling arguments. L139: why should we have 'chemical ischemia' in italic?

In the section 'Simulations and Model calibration' we explained using eqs. 5-9, how we simulate energy deprivation using $I_{\text{block}}(t)$, the parameter P_{scale} and the initial extracellular volume fraction α_e^0 for the reader to better understand the simulation results presented ahead. The data from Ref. 8 is simply used to calibrate our comprehensive model to data, and is not the driving force behind our modeling and simulations. Hence we chose to focus on the equations instead. In line 148, 'chemical ischemia' is now in quotes to highlight the specific usage of the word in context to the experimental protocol uniquely used in Ref. 8.

7. Why your model's fit/calibration in Fig. 3, Na+ traces of astrocytes and neurons have such a different shape from experiments? What contributes to it in your model? Please provide a brief account for it in the text and the figure caption.

Thank you for this comment. As suggested, we have added a brief explanation (**line 152-154**:

'*As details about the change of NKA activity resulting from chemical ischemia used in the experiments are not completely known, we observe some differences in neuronal and astrocyte* Na^+ *gradients during the onset of ED. In neurons, the presence of voltage-gated* Na^+ *channels that are activated upon membrane depolarisation causes a fast and strong* Na^+ *influx.*'

At the end of the caption of Fig. 3, we further added:

'*The qualitative difference in* Na^+ *increase between neurons and astrocytes is attributed to the presence of fast* Na^+ *influx through voltage-gated* Na^+ *channels in neurons, which are lacking in astrocytes. Please note that scaling axis in panels B and C may slightly differ between experiments and simulations for optimal display purposes.*'

Results:

There is some mess with your figures, starting from Figure 6. I have not sorted it out frankly, but it seems that you are missing at least one figure (some hypothetical Figure 9. Your current Figure 9 is what you dub as Figure 7 in the manuscript). Moreover, you are discussing results in a confusing way that does not reflect figures' numbers (e.g., Figure 6B after Figure 7; Figure 7(9) after Figure 8, frankly I got lost). I would advise using the abbreviation ED for 'energy deprivation' throughout the text.

We apologize for this mistake. The figure numbers have been corrected in the new submission. Also, energy deprivation is now abbreviated to ED throughout the text, after its first appearance in the 'Introduction' section.

- L159,161, 174: 'functional', 'fully functional' and 'non-functional' astrocyte are ambiguous.

  We removed the word 'fully' at those places to resolve the ambiguity.

- L170: I do not understand 'override.'

  Here, the word 'override' can be interpreted as 'is higher than'. We have switched to the latter phrase for clarity. We also edited line 190 to avoid another usage of that word.

- L172: It seems that your result here depends on the transients' analysis, so I do not understand why you are omitting to show it in the figure.

  We agree with the comment about transients and have updated the figure, along with other suggestions made ahead. The new Fig. 4 shows transients and the convergence to equilibrium, for both cases.

- L188: it is a bit hard to catch bistability. As I suggest below, probably transposing your figure improves readability, but the primary notion here that you are failing to state is that before and after ED, you get different steady-state solutions

We agree. The new Fig. 4 shows the two equilibria explicitly and demonstrates the existence of two, stable equilibria at baseline. We have changed **lines 194-202** to:

'*Upon restoring astrocytic function, the neuron and astrocyte do not return to their initial resting membrane voltage (Fig. 4-B). Membrane potentials remain at approximately -30 mV, and input currents can no longer trigger action potentials. From the two simulations performed Fig. 4-A and B, we see that two stable resting states are possible, i.e., the system displays bistability. The first state corresponds to membrane potentials of approximately -65 mV (neuron) and -80 mV (astrocyte), respectively, with functional synaptic transmission, i.e. the physiological state. The second corresponds to a pathological state with non-functional neurons and astrocytes, and impaired synaptic transmission.*'

- L208: what is "negligible" for you? Why?

  The volume changes are less than 5% of baseline and are hence negligible in context to pathological damage in the real brain. Comparing to the pathological case, the volume changes up to 20% of baseline and remains at that level even after energy is restored, which is indicative of cytotoxic edema, see Ref. 12. This is now made explicit as follows (**lines 219-220**):
  '*Changes in cell volume are below 5%, i.e., they are negligible for this simulation.*'

- L213,219,227: Figure number is missing.

  Our apologies, the references were made to the supplementary figures, they have been fixed in the new submission.

- L249-251: It is a good result. Link with some relevant reference and re-discuss it in the discussion section too.

  Thank you for your comment. We note that our bifurcation diagrams and discussion section 'Ischemic vulnerability depends on the extracellular volume fraction' follows your suggestion already.

- L279: Why "biophysically unrealistic"?

  We are thankful for this question as we could have been more precise. We meant to say that compared to the healthy equilibrium the sigmoidal terms are saturated at the pathological one. Then, the NKA needs to be even more active to destabilize the pathological state, i.e. the maximal activity $P_{\min}$ should be increased. Increasing this parameter to 145% of baseline behaviour is biophysically unrealistic, as there are no therapeutic interventions that enable such behaviour. To clarify this, we have adapted $I_{\mathrm{NKA}}^{\max}(t)$ to $P_{\min}$. We now write at (**lines 287-290**):

  '*As the pump is already fully activated, this resting state would become unstable only if its maximal strength $P_{\min}$ is increased above 145% of its initial value, at which a Hopf bifurcation occurs (inverted triangle), which is biophysically unrealistic.*

- Your claims concerning steady-state values are poorly substantiated based on what you show in Figs. 4, S1, and 5. In Figure 5, for example, the Volume does not seem to be at a steady-state. Improve results and substantiate your claims by improved simulations if necessary.

  Thank you for pointing this out. Regarding Fig. 4, we realised that we had started the simulation from an unstable equilibrium, which meant that the (stable) steady state that we obtain post ED was a different one. We now start the simulation from the stable steady state. Doing so ensures that post ED, the system recovers back to the original pre-ED steady state. Figs. 4 and 5 have now been updated to reflect these changes, and substantiate our claims about steady states.

- L264-311: I lost you. You are jumping back to Figure 6B, then refer to Figure 7B, very confusing. Rewrite, and rearrange figures accordingly (see also my related suggestions). Noteworthy is that In Fig.6, you consider $\alpha_e = 20\%$ vs. 80%. In Fig.7 instead you refer to $\alpha_e = 20\%$ vs 98%. Please be consistent. Moreover, 98% seems a bit extreme as a case study.

  We thank the reviewer for indicating these issues. We have updated the text, and we agree that 98% was too extreme. We have replaced the bifurcation diagram for $\alpha_e = 98\%$ by a diagram for $\alpha_e = 80\%$, see the new Figure 7A(right). We note that the latter is very similar to the previous diagram.

- L334-348: I am missing Figure 9; hence I cannot comment on this section.

  We apologize for the confusion created by figure numbers and captions. We have corrected this.

Discussion

Overall, you are failing to say what's new by your model. This is arguably in line with the fact that the research question in the introduction is not clearly stated. Some of the results also appear not to be discussed appropriately, like those in the 'Pharmacological Blockers' section, which could be linked with insights on useful drug targets.

We regret that we give that impression. We hope that our revision, curated in accordance with your suggestions, presents a clearer view of the goals. We also found that blocking voltage-gated K^+ channels helps in recovery from ED, so we added the following text to line **453-456**:
'*Our prediction regarding voltage-gated* K^+ *channels is novel with respect to current modelling literature. An experimental study showed that using the* K^+ *channel blocker tetraethylammonium (TEA) was able to attenuate ischemia-triggered apoptosis in neurons[54].*'
We present additional considerations about other channel blockade simulations and have added a few more lines (**lines 459-467**) at the end of the Discussion section on 'Neurons can be rescued from the pathological equilibrium state' to focus on the results from 'Pharmacological blockers'.
'*Decrease in NKA activity in neurons and astrocytes creates an imbalance of* Na^+ *and* K^+ *gradients, causing net compartmental* Na^+ *influx and* K^+ *efflux. In that context, it is not too surprising that our model predicts that blocking major* Na^+ *and* K^+ *pathways in the neuron presents a potential pathway for recovery from the pathological state post ED. However this is not the case for astrocytes. Blocking any of the* Na^+ *or* K^+ *pathways post ED in the astrocyte (see supplementary Figure S3 Fig) does not perturb the pathological state. This can be attributed to the fact that in our model, there is no astrocyte* Na^+ *influx or* K^+ *efflux process that contributes to large gradients, compared to the neuronal voltage-gated channels.*

1. L391-400: Overtly technical. Simplify and emphasize oscillations instead.

   The paragraph between lines 391-400 (now **419-429**) was reformulated to focus first on the experiments of Brisson and Andrew. Then, we briefly discuss the shift of the Hopf bifurcation and continue discussing its implications on the difference in recovery observed in different parts of the brain. Thus, **lines 420-424** read:

   '*The neuron makes a few transient oscillations before returning to baseline, which was indeed observed in [32]. In this case, the Hopf bifurcation (inverted triangle) in Fig.7-A shifts further to the left. Increasing baseline pump strength makes the physiological state the only stable resting state at baseline by moving the position of the Hopf bifurcation.*'

2. L439-456: These limitations can only be briefly mentioned as an extra paragraph appended to the 'Relation to other computational models.'

   Thanks for this comment. However, we we think an explicit subsection Limitations is appropriate (and commonly used). Therefore, we kept this subsection. We made the limitations more concise and added a subsection 'Future directions'.

3. You should instead replace 'Limitations' with 'Future directions' or 'Open questions.' In particular, there are three topics that you have not discussed, but you should briefly mention and refer to: the first is the fact that your ED framework is intrinsically linked with the glutamate-glutamine cycle and possibly with lactate shuttling. Additionally, these processes are inherently linked to bicarbonate ions and pH regulation. It is true that currently, you do not consider pH regulation, but the fact that you are including sodium and chloride homeostasis brings in bicarbonate trafficking too. There are a couple of recent references you may look at De Pitta in Encyclopedia of computational Neuroscience 2020; and Theparambil et al. (Rusakov and Gourine, as senior authors), Nature Communications, 2020.

   We followed this suggestion and added a new discussion section on 'Future directions' addressing the points raised.

Equations and notation:

1. E1: the variable q refers to multiple kinetic variables: you should subscript it accordingly and then refer to the supplementary material. Personally, there is no point in introducing ODE systems like 1 if you do not put in relief at least the functional coupling between the relevant variables.

   Thank you for this suggestion. We, however, choose not to follow the reviewer's comment as the variables q only represent the Hodgkin-Huxley gating variables m, h and n. The other 'kinetic' variables that are part of the glutamate recycling scheme, namely $N_{D,R_1,...}$ are referred to separately in Eq. 2.

2. You seem to interchangeably use P_{min} and P_{min} (e.g. eq. 5 and L255 vs. Table 2 or L246). Please be consistent in your notation.

   The inconsistency in the usage of P_{min} is now fixed. We stick to using P_{min}.

Figures:

Your figures are part of the manuscript that requires the most work. They need substantial improvement, as most of them are lousy in their organization and lack clarity.

1. Figure 1. Improve readability, adding what ions are transported/pass through the different elements. Why is water flux by a different arrow? It is mediated by AQPs in general, which are themselves proteins like the other elements. The shading of pre/peri-synaptic compartments is barely distinguishable. Use "Extracellular Space" instead of ECS.

   Thank you for your constructive suggestions. We have added ions associated with each nontrivial ion transport mechanism to the Figure. We also changed water flux symbols as suggested but note here that the mechanisms of water transport across cell membranes are under debate; it may reflect passage through ion channels (e.g. AQ4) and/or passage along with ion transport (e.g. NKCC1). ECS has also been changed to 'Extracellular Space'.

2. Figure 2. Merge with Figure 1, and also specify ions for all arrows. Use another box to zoom into the synapse model. It is better to redraw this model in a circular fashion (curvilinear arrows) to convey the notion of vesicular recycling better. "F" state variable then is at the bottom center, and from there, you work around the vertices of a pentagon for F, R, N, D, and I, yet using curvilinear connections. Be consistent in your notation: It is either Ca2+ or [Ca2+]n.

   Thank you for your suggestions. We, however, prefer to keep the figures split for better readability. We also prefer to stick to the current presentation of the recycling scheme flowchart. There is a difference in $k_2([\mathrm{Ca}^{2+}]_n)$ and $k_3[\mathrm{Ca}^{2+}]_n$, the former is a function dependent on $[\mathrm{Ca}^{2+}]_n$ and the latter is a scalar coefficient multiplied by $[\mathrm{Ca}^{2+}]_n$. Details are available in supplementary information.

3. Figure 3. Use full axes, not mini-axes: this will improve readability and comparison. Use the same scales for both experimental and model data. In this figure, and all the following: there are more letters than A and B in the Latin alphabet. Do not hesitate to use them to label panels in your figures properly. In this figure: rearrange the material vertically: panel A on top center. Then, panel B is in two columns (explain to me what the gray curves are). And then C for Na+ and K+ in the extracellular space. Use the same figure size for these data as in B, arrange Na+ and K+ in separate rows, and ease comparison.

   Thank you for your comment. The axes used in Fig. 3 and Fig. 8 mimic the axes used in the related experimental studies, and, for consistency, we prefer to keep it that way. We added an extra label C to separate extracellular traces from the intracellular ones. However, transposing the figure as suggested creates more white space in the figure than before. In order to avoid this, we prefer keeping the orientation as is.

4. Figure 4. Transpose. Rather than two rows, make two columns, and stretch the width of individual plots if possible. Use colored labels with subscripts rather than titles, and for colored axis tick labels, also use colored axes and ticks. There is no point in presenting results that refer to transients if you do not show them. Modify figures accordingly. Also, B plots are all in blocked conditions, and the big red box is hideous. Improve aesthetics, please. Also, use proper axes ticks. You have a massive space after 10 mins, for example. Same case for some of the y-axes. Set 3-5 axis ticks in all axes, please. Append legend at an ideal position for different line colors.

   We followed this suggestion and we transposed and stretched the figures and showed more of the transients. We also added a legend. The red box has now a lighter shade.

5. Figure 5. Same observations as in Figure 4. Again: each row can be conveniently labeled by a letter, and you can refer to them much more quickly in the text: it saves the reader's time to identify what plot you are referring to by the need to read axes labels/titles.

   Thank you for this suggestion, which we followed. We made changes to the orientation of the figures, and now show more transients and a legend. We also added labels.

6. Figures 6A and 6B should be presented separately. 6B could maybe be lumped with your current Figure 9 (for OGD) or 7B together with 8? As I said, this part of your results was not readable. 6A use circled numbers as you do in Figure 8 to refer to point to left plots and avoid crowding of unnecessary arrows. Also, I would flip the panels: heatmap on the left and plots on the right. Associate title with color bar. I do not understand arrows in axis labels. I do not understand the "Extreme energy deprivation" label. Figure 6B: the middle table is not explained. If you retain it, put x label on top. Do we need arrows?

Thank you for this suggestion. We, however, think that it is clearer to leave the grouping of the figures as they are, as Figure 8 serves as a comparison to previously performed experiments, while Figure 6 shows results while changing α_e, P_{min} and P_{scale}. This distinction helps to highlight the relation with experiments better, in our view. As we refer to the simulations 6A1 and 6A2 first, we also prefer to keep those simulations on the left. We removed the labels 'Extreme energy deprivation' and 'Smaller ECS' that were meant to indicate directions of increasing energy deprivation and decreasing extracellular volume fractions respectively. The mid table in Figure 6B is now explained in the caption, and the arrows are removed.

7. Figure 7: This figure is entirely unreadable. Again rearrange material vertically. Figure 7B seems instead related to figure 8. Concerning your bifurcation plots: Avoid zooming inboxes, and instead enlarge main axes to make diagrams larger and more readable. Choosing different colors is a debatable idea, but if you keep them, append legend. Plot trajectories in separate plots and not within the bifurcation diagram. I am also not sure I understand those trajectories. It seems that P_{min} is changing from your plots, but in the caption, P_{min} is said to be fixed. Regardless, it is confusing to map a trajectory on a bifurcation diagram, where the x-axis is a parameter. Make two new plots for $P_{min}(t)$ or whatever you are changing in time to obtain the Volume (t) in the bottom plots.

We apologize. We have revised the figure in order to avoid confusion. We have removed the transients from the bifurcation diagram, and refer only to the transients shown below the diagram in panels A1b and A2b.

8. Figure 8: please mark B1 and B2 as boxes accordingly, and append on the bottom of A, as two panels of half-width the x-axes in A. Merge with current Figure 7B (and perhaps 6B). Again, the material as I got it seems messed up.

Thank you for this suggestion. We, however, prefer to have Fig. 9 (number as per caption, we apologize for the confusion) separate from the figure that discusses the experiments from Brisson and Andrew, in order to specifically highlight the influence of blocking voltage gated Na^+ channels after the system reaches a pathological state, and the influence it has on glutamate transients. Combining it with Fig. 8 might cause confusion regarding mechanisms of recovery. Fig. 8 shows recovery with stronger baseline NKA, while Fig. 9 shows recovery after reaching the pathological state by blocking neuronal voltage-gated Na^+ channels.

Figure/Table captions.

1. Figure 1. The largest ATP consume in the presynaptic neuron, and the astrocyte is by NKA.

The corresponding line in the caption was changed to:

'*The largest ATP consumption in the presynaptic neuron and the astrocyte is by the* Na^+/K^+-*ATPase (NKA).*'

2. Figure 2. This is not a caption. Provide essential model description details accordingly in the new version (merged with figure 1).

Thank you for this suggestion. As mentioned in the section on Figure comments, we prefer to separate the figure on the glutamate recycling scheme to clearly highlight the mechanism of recycling in our model. However, we added the following statements to link the model figure and the flow-chart better. The caption now reads:

'*Glutamate recycling scheme, inspired by combining vesicle-based models from [42] and [43]. (Left) Closer view of the model scheme at the synapse and (Right) the glutamate recycling scheme. Inactive neuronal intracellular glutamate (I) moves to the depot (D) from where it is packed into vesicles which pass through five stages (N,R,R1,2,3) before they are released into the synaptic cleft (F). These stages have fast time-constants that depend on intracellular* Ca^{2+} *concentration. The stages* R_i *correspond to vesicles that are bound by i* Ca^{2+} *ions. The time-constants change when there is influx of* Ca^{2+} *in the presynaptic terminal in response to membrane depolarization. Released glutamate in the cleft can be taken up by astrocytes or back to neurons using excitatory amino acid transporters (EAATs) or leak channels, thereby recycling the released neurotransmitter.*'

3. Figure 6. you compare "large" ECS with "realistic" ECS. I do not understand your concept of "realistic." Try to be as much as possible consistent with your text: if you use physiological vs. pathological, for example, or normal vs. swelling/shrunk, then adopt the same terminology throughout the whole manuscript.

Thank you for this comment. In this context, large or small extracellular sizes correspond to initial ECS volume ratio (in %) with respect to the total volume of the system. 'Realistic' ECS sizes correspond to ∼ 20% of the total volume, while 'Large' ECS sizes would be around 80%.

References:

L 41: reference 20 should be replaced by De Pitta, Chapter 10 in Computational Glioscience 2019.

The reference has been changed as suggested.

Bibliography:

Please review your bibliography entries: there is an eclectic style: some entries are in capitalized for each word vs. others that use only sentence's capitalization. Be consistent in reference/bib style, please.

We thank the reviewer for the remark, and have changed some of the bibliography entries for consistency.

Spelling and grammar issues:

I would advice to use Grammarly for a thorough spell/grammar check upon re-submission. Please do so. In addition mind the following typos and grammar errors:

1. L33: inter-relationship → interaction;
2. L38: elucidated with... calibrated with... → by ... by ...;
3. L105-108: channels/cotransporters and currents/fluxes is confusing. Simplify accordingly.
4. L109: A short summary.
5. L123: neuronal synaptic compartment.
6. L133+ (last line): The U-shaped function I_{NKA}... it Is actually I_{block} that is U-shaped...
7. L197: unnecessary italic.
8. L219,382: Furthermore, ...
9. L220-221: cell swelling is observed (approximately...).
10. L225: This is in contrast to the scenario discussed in Fig. 5A for... where...
11. L227: S2 Fig... correct accordingly.
12. L229: depth → amount
13. L231: ... the pump's strengh.
14. L232: Using our model, We explore...
15. L253: wrong figure reference.

Thank you for pointing out these issues. We have scrutinized the text using Grammarly. Based on this, we have not implemented items 1,2,5,8,12,13. For the others, we have made the following changes: 3. changed "/" to "and", 4. short was deleted, 6. U-shaped was moved to I_{block}, 7. italics removed, 9. done, 10. done, 14. done, 15. done.

General recommendations:

In the next version, please lump all figure captions and tables to the end, providing the main body of the manuscript without interruptions. Be careful when you re-submit. The impression I got from your current manuscript is that you submitted it quickly and without too much care, as if you were coming from rejection from another journal, and you promptly rearranged the material for PCB. Whether or not this is the case, it does not play in your favor to submit a manuscript to PCB with messed-up figure's numbers and missing figures, as is the case of the present version.

We regret that we gave this impression. We made sure this revision was carefully prepared. Furthermore, we have made additional textual changes throughout to improve readability and corrected remaining typos.

We would like to thank you once more for your thorough reading and constructive comments and suggestions.
